# Predictors and Outcome of Hypoxemia in Severely Malnourished Children under Five with Pneumonia: A Case Control Design

**DOI:** 10.1371/journal.pone.0051376

**Published:** 2013-01-08

**Authors:** Mohammod Jobayer Chisti, Mohammed Abdus Salam, Hasan Ashraf, Abu S. G. Faruque, Pradip Kumar Bardhan, Abu S. M. S. B. Shahid, K. M. Shahunja, Sumon Kumar Das, Tahmeed Ahmed

**Affiliations:** 1 Centre for Nutrition & Food Security, International Centre for Diarhoeal Disease Research, icddr,b Dhaka, Bangladesh; 2 Clinical Service, icddr,b, Dhaka, Bangladesh; 3 Research Administration Services, icddr,b, Dhaka, Bangladesh; University of Liverpool, United Kingdom

## Abstract

**Background:**

There is lack of information in the medical literature on predictors of hypoxemia in severely malnourished children with pneumonia, although hypoxemia is common and is often associated with fatal outcome in this population. We explored the predictors of hypoxemia in under-five children who were hospitalized for the management of pneumonia and severe acute malnutrition (SAM).

**Methods:**

In this unmatched case-control design, SAM children of both sexes, aged 0–59 months, admitted to the Dhaka Hospital of the International Centre for Diarrhoeal Disease Research, Bangladesh (icddr,b) with radiological pneumonia and hypoxemia during April 2011 to April 2012 were studied. SAM children with pneumonia and hypoxemia (SpO_2_<90%) constituted the cases (n = 37), and randomly selected SAM children with pneumonia but without hypoxemia constituted controls (n = 111).

**Results:**

The case-fatality was significantly higher among the cases than the controls (30% vs. 4%; p<0.001). In logistic regression analysis, after adjusting for potential confounders such as nasal flaring, head nodding, inability to drink, and crackles in lungs, fast breathing (95% CI = 1.09–13.55), lower chest wall in-drawing (95% CI = 2.48–43.41), and convulsion at admission (95% CI = 3.14–234.01) were identified as independent predictors of hypoxemia in this population. The sensitivity of fast breathing, lower chest wall in-drawing and convulsion at admission and their 95% confidence intervals (CI) to predict hypoxemia were 84 (67–93)%, 89 (74–96)%, and 19 (9–36)% respectively, and their specificity were 53 (43–63)%, 60 (51–69)% and 98 (93–100)% respectively.

**Conclusion and Significance:**

Fast breathing and lower chest wall in-drawing were the best predictors of hypoxemia in SAM children with pneumonia. There thus, in resources poor settings where pulse oximetry is not available, identification of these simple clinical predictors of hypoxemia in such children could be reliably used for early O_2_ supplementation in addition to other appropriate management to reduce morbidity and deaths.

## Introduction

Hypoxemia is common in pneumonic children, which is often associated with fatal outcome [1], [2], [3]. Although severe acute malnutrition (SAM) is a common co-morbidity in under-five children with pneumonia and associated with 15 times the risks of death compared to children without severe malnutrition [4], little is known about the role of hypoxemia in such population. Pulse oximetry is the most reliable, non-invasive, accurate bedside test for measuring arterial hemoglobin oxygen saturation (SpO_2_) to assess hypoxemia [2]. This is helpful in preventing unnecessary oxy-therapy, particularly in resources constrained settings and when health workers lack confidence in using clinical parameters in assessing the need for oxygen supplementation [5]. Many health facilities in developing countries where case-fatality from pneumonia is high, including Bangladesh, experience limited availability and supply of oxygen [1], [3], [6], [7], [8] and they rarely have pulse oxymeter [1], [3]. The clinicians in such setups rely on the clinical signs of hypoxemia in pneumonic children in determining the need for oxygen supplementation. Data from the developing countries including Bangladesh revealed that the clinical signs of hypoxemia, even in well nourished children with pneumonia, are often insensitive [3], [5], [6]. Like other hospitals of Bangladesh and in other developing countries, the case-load of SAM children with pneumonia is high at the Dhaka Hospital of icddr,b [9], [10]. However, there is no published data on the clinical predictors of hypoxemia in SAM children with pneumonia. Identification of predictors of hypoxemia in such population may help prudent use of oxygen therapy leading to reduces morbidity and deaths in resource-poor settings. Thus, the aims of our study were to identify the prevalence, clinical predictors and outcomes of hypoxemia in hospitalized, under-five SAM children with pneumonia.

## Materials and Methods

### Ethics statement

The study (protocol number: PR-10067) was approved by the Research Review Committee (RRC) and the Ethical Review Committee (ERC) of icddr,b and an informed written consent was obtained from parents or guardians of all participating children. Children whose caregivers did not give consent were not included in the study.

### Study design

The study was conducted at the Dhaka Hospital of icddr,b. We used an unmatched case-control design and enrolled 334 children of both sexes, aged 0–59 months, who were admitted to the Intensive Care Unit (ICU), High Dependency Unit (HDU), or Acute Respiratory Infection (ARI) Ward of the hospital between April 2011 and April 2012. SAM children with radiological pneumonia and hypoxemia (n = 37) constituted the cases, and SAM children with radiological pneumonia but no hypoxemia constituted the controls. Controls were randomly selected by computer randomization using SPSS (version 17.0; SPSS Inc, Chicago) from a personal computerized data source of this study. This database identified 297 controls, and 1∶3 unmatched case-control ratio was used to increase the statistical power of our analyses. We defined hypoxemia based on WHO recommendations (SpO_2_<90%) [11]. Pneumonia was diagnosed based on radiological evidence of consolidation or patchy opacities [10] and the interpretation of CXR for the diagnosis of pneumonia was initially done by at least two clinicians including principal investigators (MJC) and finally confirmed by a qualified radiologist of the Dhaka Hospital of icddr,b. Children with severe wasting [z score for weight for height<−3 of the median of the WHO anthropometry] or severe under-nutrition [z score for weight for age<−4 of the median of the WHO anthropometry], or nutritional edema were considered as severe malnutrition.

### Setting

The Dhaka Hospital of icddr,b provides care and treatment to around 140,000 patients of all ages and both sexes with diarrhea, with or without associated complications and with or without other health problems. Diarrhea and/or acute respiratory infection (ARI) is the entry point for admission to icddr,b. Patients with complications of diarrhea, or those with respiratory distress, cyanosis, apnea, hypothermia, sepsis, shock, impaired consciousness, convulsion, severe/very severe pneumonia with hypoxemia or respiratory failure are admitted to the ICU. Children with severe pneumonia and hypoxemia are admitted to the high dependency unit (HDU) and those with severe pneumonia but no hypoxemia are admitted to the ARI ward. The vast majority of the patients at icddr,b have poor socio-economic backgrounds and live in urban and peri-urban Dhaka.

### Patient management

Patients admitted to the ICU/HDU/ARI ward receive care and treatment, which includes antibiotic therapy, supportive care such as intravenous fluids and oxygen, frequent monitoring, and nutritional support (breast milk, micronutrients, zinc). Mechanical ventilation is used for management of children admitted to ICU with respiratory failure and children irrespective of presence or absence of SAM have equal access to ICU/mechanical ventilation. All children in the study were assessed by the duty physician, who took medical history, performed clinical examinations, and determined management plan. Arterial oxygen saturation (SpO_2_) was measured using a portable pulse oximeter (OxiMax N-600, Nellcor, Boulder, CO) and blood glucose was estimated using a Gluco-check machine (STADA, Bad Vilbel, Germany).

Children with hypoxemia received O_2_ supplementation through nasal prongs (2 L/min) or mask (5 L/min). Antibiotics were prescribed for children with pneumonia, sepsis, severe cholera, dysentery, severe malnutrition, and other bacterial infections. Dehydration was corrected using ORS solution, orally or through NG tube, or appropriate intravenous fluid when severely dehydrated or when they had severe respiratory distress. Pneumonia was managed according to the WHO algorithm [19] and management of severe protein-energy malnutrition was done followed the hospital's guidelines [19], [20].

### Measurements

Case report forms (CRF) were developed, pretested, and finalized for data acquisition. Characteristics analyzed include demographic (age, gender, stopped breast feeding at neonatal period), clinical signs [age specific fast breathing (respiratory rate ≥60/minute in children <2 months of age, respiratory rate ≥50/minute in children 2 months to <12 months of age, and respiratory rate ≥40/minute in children 12 months to 59 months of age), lower chest wall in-drawing (inward movement of the bony structures of the lower chest wall with inspiration), nasal flaring, head nodding, crackles in lungs (by auscultation), dehydration (defined by “Dhaka methods” of assessment of dehydration that is almost similar to WHO method and approved by WHO [12]), convulsion at admission, inability to drink and temperature], clinical diagnosis [severe sepsis (defined by sepsis plus absent peripheral pulses or capillary refilling time ≥2 second or hypotension)] [3], [13], and outcome.

### Analysis

All data were entered into SPSS for Windows (version 15.0; SPSS Inc, Chicago) and Epi-Info (version 6.0, USD, Stone Mountain, GA). Differences in proportions were compared by the Chi-square test. In normally distributed data differences of means were compared by Student's t-test and Mann-Whitney test was used for comparison of data that were not normally distributed. A probability of less than 0.05 was considered statistically significant. Strength of association was determined by calculating odds ratio (OR) and their 95% confidence intervals (CIs). In identifying predictors of hypoxemia in children with SAM and pneumonia, variables were initially analyzed in a uni-variate model, then independent predictors were identified using logistic regression after controlling co-variates. We also calculated the sensitivity and specificity, and positive and negative predictive values of the independent predictors of hypoxemia.

## Results

The prevalence of hypoxemia in pneumonia children with SAM at the Dhaka Hospital of icddr, b during the study period was estimated at 11% (37/334). The sex and age distribution among the cases (hypoxemic SAM children with pneumonia) and controls (non-hypoxemic SAM children with pneumonia) groups were comparable, and so were the proportion of children who stopped breast feeding in neonatal period, temperature, severe sepsis, hypoglycemia and serum magnesium (Table 1). The case-fatality was significantly higher among the cases (hypoxemic SAM children with pneumonia) than among the controls (non-hypoxemic children), and they more often had higher serum sodium compared to the controls (Table 1). In logistic regression analysis, after adjusting for potential confounders such as nasal flaring, head nodding, inability to drink, and crackles in lungs, fast breathing, lower chest wall in-drawing and convulsion at admission were retained as independent predictors of hypoxemia (Table 2). The sensitivity, specificity, positive predictive value (PPV) and negative predictive value (NPV) of fast breathing, lower chest wall in-drawing, convulsion at admission, nasal flaring, head nodding, unable to drink, and crackles in lungs with their 95% confidence intervals to predict hypoxemia are shown in Table 3.

**Table 1:** Characteristics of the hypoxemic under-five children with pneumonia and SAM admitted to the ICU/HDU/ARI ward of the Dhaka Hospital of icddr,b.

Characteristic	Hypoxemia (n = 37)	Normal oxygen saturation (n = 111)	OR (95% CI) (unadjusted)	p
Male	17 (46)	84 (53)	0.75 (0.33–1.68)	0.569
Age (months) (median, IQR) (range)	6.2 (3.5, 11.2) 0.5–48	8.0 (4.5, 14.0) 0.7–59	-	0.182
Stopped breast feeding at neonatal period	13 (35)	33 (30)	1.28 (0.54–3.02)	0.682
Convulsion at admission	7 (19)	2 (2)	12.72 (2.24–93.99)	<0.001
Lower chest wall indrawing	33 (89)	44 (40)	12.56 (3.87–45.13)	<0.001
Age specific fast breathing	31 (84)	52 (47)	5.86 (2.11–17.09)	<0.001
Crackles in lungs	31 (84)	74 (67)	2.58 (0.92–7.60)	0.076
Nasal flaring	2 (5)	1 (1)	6.29 (0.43–180.95)	0.154
Head nodding	2 (5)	1 (1)	6.29 (0.43–180.95)	0.154
Inability to drink	5 (14)	11 (10)	1.42 (0.39–4.89)	0.548
Temperature (° Celsius) (mean ± SD)	37.6 ±1.4	37.8 ±1.2	-	0.227
Severe sepsis	10 (27)	19 (17)	1.79 (0.68–4.68)	0.282
Hypoglycemia (RBS<3.0 mmol/L)	1 (3)	1 (1)	3.06 (0.0–115.25)	0.439
Serum sodium (mmol/L)	143.5 ±16.6	136.8 ±16.6	-	0.027
Serum magnesium (mmol/L)	1.1 ±0.2	1.0 ±0.3	-	0.562
Outcome (Died)	11 (30)	4 (4)	11.32 (2.99–46.41)	<0.001

Figures represent n (%) unless indicated otherwise.

OR = Odds Ratio; CI = Confidence Interval; IQR = Inter quartile range; SD = Standard Deviation; RBS = Random Blood Sugar.

**Table 2:** Results of logistic regression to explore the independent predictors of hypoxemia in under-five SAM children with pneumonia.

Predictor	Adjusted OR	95% CI	p
Fast breathing	3.84	1.09–13.55	0.036
Lower chest wall in-drawing	10.38	2.48–43.41	0.001
Convulsion at admission	27.12	3.14–234.01	0.003
Nasal flaring	5.71	0.28–115.03	0.256
Head nodding	7.72	0.53–112.99	0.136
Crackles in lungs	0.47	0.12–1.85	0.280
Inability to drink	1.35	0.58–3.15	0.481

**Table 3:** Sensitivity, specificity, positive and negative predictive value of predictors of hypoxemia in the severely malnourished children with pneumonia.

Variable	Hypoxemic (n = 37)	Non-hypoxemic (n = 111)	Sensitivity (95% CI)	Specificity (95% CI)	PPV (95% CI)	NPV (95% CI)
Fast breathing	31 (84)	52 (47)	84 (67–93)	53 (43–63)	37 (27–49)	91 (80–96)
Lower chest wall in-drawing	33 (89)	44 (40)	89 (74–96)	60 (51–69)	43 (32–55)	94 (84–98)
Convulsion at admission	7 (19)	2 (2)	19 (9–36)	98 (93–100)	78 (40–96)	78 (70–85)
Nasal flaring	2 (5)	1 (1)	5 (1–20)	99 (94–100)	67 (13–98)	76 (68–82)
Head nodding	2 (5)	1 (1)	5 (1–20)	99 (94–100)	67 (13–98)	76 (68–82)
Crackles in lungs	31 (84)	74 (67)	84 (67–93)	33 (25–43)	30 (21–39)	86 (71–94)
Inability to drink	5 (14)	10 (10)	14 (5–30)	90 (83–95)	31 (12–56)	76 (67–83)

Figures represent n (%) unless indicated otherwise.

PPV = Positive predictive value; NPV = Negative predictive value.

## Discussion

We observed fast breathing, lower chest wall in-drawing, and convulsion at admission as independent predictors of hypoxemia in under-five SAM children with pneumonia whereas in exception of lower chest wall in-drawing, different clinical predictors of hypoxemia in a recently published study in under-five children involving mostly well nourished children from the same country were observed [3]. In pneumonic children with fast breathing, hypoxemia may occur as a consequence of impairment of alveolar-arterial oxygen diffusion and concomitant increase in the partial pressure of carbon-dioxide (CO_2_) due to abnormally lower alveolar ventilation [14]. The sensitivity, specificity, NPV and 95% confidence intervals (CI) of fast breathing and lower chest wall in-drawing in hypoxemic SAM children with pneumonia were comparatively better than those observed in a recently published study [3] from the same country that was done in children mainly involved pneumonia without severe malnutrition. Fast breathing and lower chest wall in-drawing would normally be lesser expected in pneumonia in severely malnourished children might be due to reduced strength in accessory muscles activity related to supporting ventilation, consequent to their reduced total body electrolytes (except sodium), particularly potassium and magnesium [15], [16]. However, these two parameters in SAM children with pneumonia and hypoxemia in our present study were frequently observed, which was beyond our expectation and this might be due to profound ventilation perfusion mismatch, potentially resulting in the rise of partial pressure of carbon-dioxide (CO_2_) leading to rapid breathing and lower chest wall in-drawing in an effort to eliminate CO_2_ from the pulmonary circulation [14]. This might also explain the higher sensitivity and NPV and reasonable specificity of fast breathing and lower chest wall in-drawing as predictors of hypoxemia in such population. Age specific fast breathing and lower chest wall in-drawing are the basis of the WHO algorithm for diagnosing clinical pneumonia in under-five children [17]. Our findings suggests that SAM children with cough or respiratory difficulty who also have any of these two indicators (fast breathing and lower chest wall in-drawing) are likely to be hypoxemic and they should receive oxygen supplementation in addition to other management of pneumonia and malnutrition, particularly in settings where pulse oximetry is not accessible due to any reason.

We also observed that hypoxemic SAM children with pneumonia attended the hospital more often with convulsions. Convulsions is a warning sign of severe/very severe pneumonia which might be related to cerebral ischemia either from hypoxemia or impaired cerebral perfusion secondary to consequence of sepsis [18]. We performed careful neurological examinations, but did not have the facilities for performing EEG. Except for higher serum sodium in cases, we did not observe any differences in the distributions of other possible factors for convulsion, such as blood glucose, serum magnesium, body temperature, severe sepsis, and CSF studies. However, normal range of median value of the serum sodium in both the groups reflects that higher serum sodium might not be clinically relevant for the development of convulsion in our patient population. Therefore, among the factors we looked for, hypoxemia remains as the known contributor to convulsion [19], [20]. With very low sensitivity and high specificity, and reasonable PPV and NPV, using convulsion as a predictor would miss out hypoxemia in 80% of SAM children with pneumonia. However, due to high specificity, presence of convulsion in SAM children with pneumonia would be an indication for oxygen therapy; our observation is similar to the findings of our earlier study in children in which we did not analyze nutritional status of the children [3].

We observed significantly higher case-fatality among hypoxemic SAM children with pneumonia. They also had higher serum sodium concentration, which is often associated with fatal outcome [19], [21]. More importantly, hypoxemia in SAM children with pneumonia likely represents very severe illness. The association of hypoxemia and death has been reported by earlier studies; however, they did not describe nutritional status of the children [22], [23].

The prevalence of hypoxemia in children with pneumonia may vary from region to region [22], [24], [25], [26]. However, ours is the first report on prevalence (11%) of hypoxemia in SAM children with pneumonia. The prevalence of hypoxemia in our study is similar to previous observation in better nourished children with pneumonia [26].

Although radiologically confirmed pneumonia used to have high specificity for pneumonia, it is also a main limitation of the study and is not generalizable to all WHO clinical pneumonia and probably accounts for less than 50% of WHO clinical severe lower respiratory tract infection [27].

In conclusion, hypoxemia is associated with high case fatality in under-five SAM children with pneumonia. Such children presenting with age specific fast breathing, lower chest wall in-drawing, and convulsion at admission are more likely to be hypoxemic. However, age specific fast breathing and lower chest wall in-drawing, the basis of the WHO algorithm for diagnosing clinical pneumonia, are the best predictors of hypoxemia among under-five SAM children with pneumonia. Thus, in resource poor settings, where pulse oximetry is not available, careful observation for these simple predictors of hypoxemia in SAM children with cough or respiratory difficulty will be critically important for initiation of early O_2_ supplementation in addition to other appropriate management in an effort to reduce morbidity and deaths.
